# Analysis of the frequency of EGFR, KRAS and ALK mutations in patients with lung adenocarcinoma in Croatia

**DOI:** 10.1186/s13000-016-0544-9

**Published:** 2016-09-21

**Authors:** Luka Brcic, Marko Jakopovic, Marija Misic, Fran Seiwerth, Izidor Kern, Silvana Smojver-Jezek, Franz Quehenberger, Miroslav Samarzija, Sven Seiwerth

**Affiliations:** 1 Institute of Pathology, Medical University of Graz, Auenbruggerplatz 25, 8036 Graz, Austria; 2 Institute of Pathology, University of Zagreb School of Medicine, Zagreb, Croatia; 3 Department for Respiratory Diseases Jordanovac, University of Zagreb School of Medicine, University Hospital Centre Zagreb, Zagreb, Croatia; 4 Cytology and Pathology Laboratory, University Clinic of Respiratory and Allergic Diseases, Golnik, Slovenia; 5 Clinical Department of Pathology and Cytology, University Hospital Centre Zagreb, Zagreb, Croatia; 6 Institute for Medical Informatics, Statistics and Documentation Medical University of Graz, Graz, Austria

**Keywords:** Lung adenocarcinoma, EGFR, KRAS, ALK, Gender, Smoking

## Abstract

**Background:**

Many studies have been published on the mutational status of patients with lung adenocarcinomas, and great population-based variability in mutation frequencies has been reported. The main objective of the present study was to analyze the EGFR, KRAS and ALK mutation status in a representative cohort of patients in Croatia with lung adenocarcinomas and to correlate the mutational status with clinical data.

**Methods:**

All patients who were newly diagnosed within 6 months with histologically proven primary lung adenocarcinomas were included. Mutational analyses for EGFR and KRAS mutations were performed in a cobas z 480 analyzer. ALK immunohistochemistry was performed using the D5F3 clone on Benchmark XT instrument. Clinical data were obtained from the medical records.

**Results:**

Of the 324 patients, 59.9 % were male. At the time of diagnosis, the patients ranged in age range from 35 to 88 years (median 63 years). Most of the patients were current smokers or former smokers (77.2 %). EGFR mutations were found in 15.7 % of the patients, and of these mutations, exon 19 deletion was the most common (45.1 %). KRAS mutations were present in 34.9 % of the patients, while 4.1 % of patients were ALK-positive. The statistical significance of the presence of mutations was detected for both gender and smoking.

**Conclusion:**

The detected mutation rates demonstrated a slightly higher prevalence of KRAS mutations, but not a higher prevalence of EGFR mutations or ALK gene rearrangement, in comparison with the rates found in other European countries. EGFR and ALK mutational status showed a statistically significant correlation with gender as well as with smoking, while KRAS mutation status showed a statistically significant correlation only with smoking.

**Electronic supplementary material:**

The online version of this article (doi:10.1186/s13000-016-0544-9) contains supplementary material, which is available to authorized users.

## Background

In the last decade, great improvements have been made in the development of targeted therapies for lung cancer. To date, all approved targeted therapies have been aimed at adenocarcinomas of the lung, with adequate targets detected. Since patients with lung carcinoma usually present in an advanced stage, and the amount of available tissue is limited, it can be very difficult to reach a diagnosis and preserve sufficient tissue for molecular testing.

Molecular testing for epidermal growth factor receptor (EGFR) and anaplastic lymphoma kinase (ALK) mutations should be a current standard in pathology practice. The prevalence of EGFR mutations varies among different populations, and these mutations are present in higher frequencies in women, in light or never-smokers and in East Asian patients [1–7]. Patients with specific mutations in the EGFR gene will respond differently to tyrosine kinase inhibitor (TKI) therapy. Patients with EGFR-mutated lung cancers have a better prognosis compared with patients without mutations, regardless of therapy type [8]. ALK gene rearrangement, which is another targetable genetic change in lung adenocarcinomas, was discovered in 2007 [9]. The frequency of ALK rearrangements is approximately 5 % in lung adenocarcinomas [9, 10] and is higher in light or never-smokers and in younger individuals [11–13]. Mutations in Kirsten rat sarcoma viral oncogene homolog (KRAS) are the most common mutations in lung adenocarcinomas, but currently, no targeted therapy is available. According to some studies, KRAS mutations are more prevalent in women, but other reports found no difference in regards to gender [14–16]. Mutations in EGFR, ALK and KRAS are generally regarded as mutually exclusive [8, 10, 11, 17].

The aim of this study was to present the EGFR, KRAS and ALK mutations in a representative cohort of patients with lung adenocarcinomas in Croatia and to correlate the mutational status with clinical data.

## Methods

### Patients

This study was was performed at the University of Zagreb School of Medicine, Department of Respiratory Diseases Jordanovac, and the Institute of Pathology, Zagreb, Croatia. All patients who underwent routine bronchoscopy procedures or surgery for the diagnosis or treatment of lung carcinoma from 1.5.2014 to 1.11.2014, as well as those who were diagnosed with lung adenocarcinomas, adenosquamous carcinomas or non-small cell lung carcinomas not-otherwise specified (NSCLC-NOS) were included. The 7th edition of the Union for International Cancer Control (UICC) TNM classification criteria were used for staging. A smoker was defined as a patient who has smoked at least 100 cigarettes in a lifetime and is still smoking, a former smoker was defined as a patient who has smoked at least 100 cigarettes in a lifetime, but is currently not smoking, and a never-smoker was defined as a patient who has never smoked or who has smoked fewer than 100 cigarettes in a lifetime. Diagnostic procedures were performed according to the proposed 2011 adenocarcinoma classification by the International Association for the Study of Lung Cancer/American Thoracic Society/European Respiratory Society [18], using additional immunohistochemistry when needed, either the residual section of formalin fixed paraffin embedded (FFPE) tissue, or May-Grünwald Giemsa (MGG) stained cytological smears or frozen unstained smears, were submitted for molecular testing.

### Mutational analyses

Samples were tested in a sequential order; first for EGFR mutations, followed by KRAS testing of EGFR-negative samples and ALK rearrangement testing of EGFR- and KRAS-negative samples.

#### EGFR and KRAS mutational analyses

DNA was extracted from FFPE tissue samples and cytological samples, and sometimes macrodissection was performed to ensure a content of at least 10 % tumor. DNA was extracted using a cobas DNA Sample Preparation Kit (Roche Diagnostics GmBH, Mannheim, Germany) according to the package inserts. The measurement of the DNA quantity was performed using a Nanodrop ND-1000 spectrophotometer (NanoDrop Technologies, Wilmington, DE, USA). Samples were tested by polymerase chain reaction (PCR) in a cobas z 480 analyzer (Roche Diagnostics GmBH, Mannheim, Germany) using the cobas EGFR Mutation Test, which can detect 41 mutations in exons 18, 19, 20 and 21 of the EGFR gene, and the cobas KRAS Mutation Test (both Mutation Tests purchased from Roche Diagnostics, GmBH, Mannheim, Germany), which can detect mutations in codons 12, 13, and 61.

#### ALK immunohistochemistry

The analysis of the tumors for ALK gene rearrangement was performed by immunohistochemistry on a Benchmark XT instrument (Ventana Medical Systems, Tucson, AZ) using the Ventana ALK antibody clone D5F3 (Roche Diagnostics GmBH, Mannheim, Germany) with the OptiView detection kit (Roche Diagnostics GmBH, Mannheim, Germany). The results were scored as either positive or negative according to the manufacturer’s recommendations.

### Statistical analysis

Associations between gender, smoking status, Eastern Cooperative Oncology Group (ECOG) performance status and mutation status were tested using Pearson’s chi square test. Associations between age at diagnosis, T stage, N stage, M stage, clinical stage and mutation status were tested using Wilcoxon’s rank sum test. Confounding risk factors were eliminated by stratification using the Cochran-Mantel-Haenszel method. Logistic regression was used in order to test for the interaction of gender and smoking status with respect to mutation status. R 3.2.3 was used for all calculations. *P* values less than 0.05 were considered statistically significant.

## Results

### Clinical data

In the designated 6-month period, all together 332 patients were newly diagnosed with adenocarcinomas (320), adenosquamous carcinomas [1] or NSCLC-NOS [11]. Of these samples, adequate material for EGFR testing was available for 324 patients, while 8 samples from patients with NSCLC-NOS were not further evaluated due to insufficient tissue or poor DNA quality. Resected tissue from 69 patients (69/324, 21.3 %) was available, while for the remaining patients (255/324, 78.7 %), biopsies/cytological samples were used. Due to sequential testing, the time from sample arrival at the molecular laboratory to EGFR mutation analysis was, on average, 8 working days. For KRAS analysis, the total turnaround time was 11 working days, and for ALK rearrangement analysis, the turnaround time was as high as 25 working days.

In all, 194 males (194/324, 59.9 %) and 130 females (130/324, 40.1 %) were included in the analysis. The age at diagnosis ranged from 35 – 88 years for males (median 62 years) and from 39 – 86 years for females (median 64.5 years). The number of smokers and former smokers was higher in the male population (170/194, 87.6 %), as only 80/130 (61.5 %) females were smokers or former smokers. The majority of the patients were in clinical stage IIIB and IV (222/324, 68.5 %), followed by stages II-IIIA (63/324, 19.5 %) and stage I (26/324, 8.0 %) (Table 1).

**Table 1:** Summary of clinical and molecular-pathological characteristics^a^

	TOTAL	EGFR		KRAS		ALK	
		wt	mut	wt	mut	wt	mut
	324 (100)						
Age (range, median)	35-88, 63						
Gender							
Male	194 (59.9)	183 (94.3)	**11 (5.7)**	97 (53.6)	**84 (46.4)**	80 (97.6)	**2 (2.4)**
Female	130 (40.1)	90 (69.2)	**40 (30.8)**	61 (67.8)	**29 (32.2)**	40 (80.0)	**10 (20.0)**
Smoking status							
Smokers	147 (45.4)	139 (94.6)	**8 (5.44)**	75 (54.3)	**63 (45.7)**	65 (95.6)	**3 (4.4)**
Former smokers	103 (31.8)	92 (89.3)	**11 (10.7)**	49 (53.8)	**42 (46.2)**	37 (97.4)	**1 (2.6)**
Never-smokers	74 (22.8)	42 (56.8)	**32 (43.2)**	34 (81)	**8 (19)**	18 (69.2)	**8 (30.8)**
Clinical stage^b^							
I	26 (8.0)	22 (8.1)	4 (7.8)	8 (5.1)	11 (9.2)	7 (5.8)	0 (0)
II-IIIA	63 (19.5)	51 (18.7)	12 (23.5)	31 (19.6)	20 (16.8)	28 (23.3)	1 (8.3)
IIIB-IV	222 (68.5)	190 (69.6)	32 (62.7)	112 (70.9)	76 (63.9)	79 (65.8)	10 (83.3)

*Legend*: ^a^data presented as absolute numbers and percentages in parentheses; *wt* wild type, *mut* mutated, ^b^ for 12 patients no data for clinical stage were available; numbers in bold indicate a statistically significant difference

### EGFR mutation analysis

In the entire group of 324 patients, 51 patients (15.7 %) had EGFR mutations. The most common were exon 19 deletions (23/51, 45.1 %), followed by the exon 21 point mutation L858R (20/51, 39.2 %), the exon 18 point mutation G719X (4/51, 7.8 %), and exon 20 insertions (2/51, 3.9 %). In addition, two patients had more than one mutation, one of which was a resistance mutation (G719X+ S768I in a male patient, a former smoker; L858R + T790M in a female patient, a never-smoker). The types of EGFR mutations are presented in Table 2. The distribution of EGFR mutations according to gender showed a high predominance in the female group, where 40/130 (30.8 %) had EGFR mutations, the most common of which were exon 19 deletions (21/40, 52.5 %). In comparison, 11/194 patients (5.7 %) in male subgroup had EGFR mutations, of which L858R was the most common (6/11, 54.5 %). The highest incidence of EGFR mutations was observed in never-smokers (regardless of gender) 32/74 (43.2 %), was much lower in former smokers, 11/103 (10.7 %) and was lowest in smokers (8/147, 5.4 %). The most common mutations in smokers and former smokers were exon 19 deletions (5/8, 62.5 % and 4/11, 36.4 %, in smokers and former smokers, respectively). The associations between the presence of EGFR mutations and either gender (*p* < 0.0001) or smoking status (*p* < 0.0007) were demonstrated to be statistically significant. In female never-smokers (50 patients), 26 had EGFR mutations (26/50, 52 %), while only seven of 57 female smokers had EGFR mutations (7/57, 12.3 %). Interestingly, of 23 female former smokers, seven female former smokers (7/23, 30.4 %) had EGFR mutations. No statistically significant differences were observed between the presence of mutations and the wild type genotype with respect to ECOG, T stage, N stage, M stage, clinical stage, or type of material used for testing. The difference between genders remained significant after stratification by smoking status (*p* < 0.0001), and the differences between smoking status remained significant after stratification by gender (*p* < 0.0001). The interaction between smoking and gender was not statistically significant according to logistic regression (*p* = 0.38).

**Table 2:** Distribution of EGFR, KRAS and ALK mutations according to gender and smoking status^a^

	Gender		Smoking status		
	M	F	S	F	N
EGFR mutations present	**11**	**40**	**8**	**11**	**32**
Exon 19 deletion	2	21	5	4	14
L858R	6	14	2	4	14
G719X	1	3	1	1	2
Exon 20 insertions	1	1	0	1	1
G719X + S768I	1	0	0	1	0
L858R + T790M	0	1	0	0	1
KRAS mutations present	**84**	**29**	**63**	**42**	**8**
Codon 12/13	79	28	59	41	7
Codon 61	5	1	4	1	1
ALK translocation	**2**	**10**	**3**	**1**	**8**

*Legend*: ^a^Data presented as absolute numbers. *M* male, *F* female, *S* smoker, *F* former smoker, *N* never-smoker, numbers in bold indicate a statistically significant difference

### KRAS mutation analysis

EGFR-negative patients (273 in all) were further tested for KRAS mutations. KRAS mutations were detected in 113 (41.4 %) patients in this “enriched” group. The great majority (107/113 patients; 94.7 %) had mutations in codons 12/13, and 6/113 (5.3 %) had mutations in codon 61 (five of these patients were smokers or former smokers). KRAS mutations were more frequent in males (84/181, 46.4 %) than in females (29/90, 32.2 %) and were very common in smokers (63/138, 45.7 %) and former smokers (42/91, 46.2 %) compared with never-smokers (8/42, 19 %), regardless of gender (Table 2). The correlation between KRAS mutational status and smoking status (*p* = 0.0053) and between KRAS mutational status and gender (*p* = 0.026) proved to be statistically significant. No statistically significant differences were found between mutations and the wild type genotype with respect to age, ECOG, T stage, N stage, M stage, clinical stage or type of material used for testing. The difference between genders was not significant after stratification by smoking status (*p* = 0.11). However, the differences between smoking status remained significant after stratification by gender (*p* = 0.016). The interaction between smoking and gender was not statistically significant according to logistic regression (*p* = 0.98).

### ALK gene rearrangement testing

ALK immunohistochemical analysis was performed in the 160 EGFR- and KRAS-negative patients. Testing was successful in samples from 82 males and 50 females. In 28 samples, there was insufficient material for further analyses. In males, 2/82 (2.44 %) had ALK gene rearrangements, while in females, 10/50 patients (20 %) were demonstrated to have ALK-positive rearrangement based on strong immunohistochemical reaction. The highest incidence of ALK gene rearrangement was observed in never-smokers (8/26, 30.8 %), followed by a very low incidence in smokers (3/68, 4.4 %) and former smokers (1/38, 2.6 %) (Table 2). These correlations between ALK status and gender (*p* < 0.0001) as well as between ALK status and smoking status (*p* = 0.0007) were also statistically significant. No statistically significant differences were observed between the presence of mutations and wild type genotype with respect to age, ECOG, T stage, N stage, M stage, clinical stage or type of material used for testing. The difference between genders remained significant after stratification by smoking status (*p* = 0.0082), and the differences between smoking status remained significant after stratification by gender (*p* = 0.0031). The interaction between smoking and gender was not statistically significant according to logistic regression (*p* = 0.52)

Since genetic changes in EGFR, KRAS and ALK are practically mutually exclusive, we can conclude that in our study group of 324 patients, 51 had EGFR mutations (15.7 %), 113/324 had KRAS mutations (34.9 %), and, of the 296 patients with enough material for immunohistochemical analysis of ALK translocations, 12 were positive (4.1 %) (Additional file 1: Table 1).

## Discussion

We presented the results of the EGFR, KRAS and ALK mutational analyses in patients with lung adenocarcinomas in a prospective study, regardless of clinical stage. The analyses were performed only on patients who were newly diagnosed during a 6-month period at a single institution in Croatia. Importantly, the Department of Respiratory Diseases Jordanovac at the University Hospital Centre Zagreb is a referral center for lung diseases, and the majority of patients with lung carcinoma (almost 60 %) are diagnosed and treated here. According to available data from the Croatian National Cancer Registry for 2014, the number of patients with newly diagnosed carcinoma of the lung was 2915, and, of those, 1693 were diagnosed in our department (1693/2915, 58.1 %) [19]. To date, this is the largest, most representative study of the mutational status of Croatian patients with lung adenocarcinomas. Our population was composed primarily of males (59.9 %) and a high proportion of smokers and former smokers (77.2 %); this population is practically identical to that of the recently published INSIGHT Study [20] and is also similar to those in other studies [21, 22].

In many published studies, a wide range in the prevalence of EGFR mutations has been reported in lung carcinomas, from 7.5 % in Norway [23, 24], 8.8 % in a mixed ethnic population in the USA [10], to 10 to 15 % in Europe [20, 22], to more than 50 % in Asian countries [25, 26]. In the study by Zaric et al., the authors detected the presence of EGFR mutations in 11.7 % of their patients [22], while the INSIGHT Study showed a prevalence of 13.8 % of EGFR mutations in patients with lung carcinomas [20]. Our prevalence of 15.7 % in EGFR-mutated lung adenocarcinomas seems to be among the higher rates in European countries and is closer to what was reported in the Russian study by Moiseyenko, where the prevalence was 19.8 % [27].

What might be the reason for such a difference? There are some very important, and usually neglected differences, between published studies in regards to patient selection criteria (clinical and pathological), as well as the methods used for mutational analysis. In a French study by Vallee et al., one of the largest studies in Europe, [28] 1403 tumor samples were analyzed, of which 1144 were adenocarcinomas and 101 were NSCLC-NOS; EGFR mutations were found in 14.7 % and 4.0 %, of adenocarcinomas and NSCLC-NOS, respectively. They used fragment analysis for exon 19 deletions and allele-specific PCR to detect the L858R mutation. Other mutations were not explored, and thus the true incidence of EGFR mutations in this population is almost certainly a bit higher than reported. Milella et al. presented the results of patients in clinical stages IIIB and IV, with histologically heterogeneous tumors (125 adenocarcinomas, 17 squamous cell lung carcinoma and 46 other lung carcinomas) [21]. They also analyzed only exon 19 deletions and the exon 21 point mutation, and reported that 9.0 % of patients had EGFR mutations, but 40.4 % of their patient population was unable to be evaluated. Even when we analyzed what was apparently the lowest reported rate of EGFR mutations (7.5 %) in a study in Norway [23], we see that, of 240 tumors, only 141 were actually lung adenocarcinomas, and only 11 % of patients had EGFR mutations. The patients included in that study all had operable lung cancers, and the investigators used a TheraScreen EGFR mutation kit (DxS, Manchester, UK) to detect 28 specific mutations; some samples were further analyzed by denaturing high-performance liquid chromatography and sequencing. In a more recent study in Norway, the prevalence of EGFR mutations in lung adenocarcinomas was found to be 9.4 % [24]. However, the INSIGHT Study, which comprises results form 6 central European countries each with different inclusion criteria and testing methods, analyzed 1785 patients, of whom 1393 were diagnosed with adenocarcinomas [20]. The prevalence of EGFR mutations in patients with adenocarcinomas was 15.4 %, which was practically identical to our findings. A Russian study analyzed only lung adenocarcinomas, which were assessed by PCR in a Cycler iQ Real Time Detection System (Bio Rad Laboratories, GmBH, Munich, Germany); the results revealed a prevalence of EGFR mutations that was higher compared with that in other European countries (19.8 %) [27], but was still lower than that in Asian populations, in which the prevalence is above 40 %. In one study in China, the authors reported EGFR mutations in 66.3 % of consecutively collected lung adenocarcinomas, which were analyzed by sequencing [25]. Another interesting point in the comparison of our results with those of the INSIGHT study was the frequency of specific mutations; while practically no differences were observed in the frequency of exon 19 deletions (the most common mutation type) and exon 20 insertions, the frequency of the L858R mutation was higher in our patients (representing 39.2 % of all mutations compared with 28.3 % in the INSIGHT Study) [20]. To date, only one study with data on EGFR mutations in a Croatian population was published. This study, by Mohar et al., reported a prevalence of EGFR mutations of 19.8 % in tested patients [29]. However, in this paper, the authors analyzed only cytological samples that were obtained from more than one hospital, and it is not clear from the paper whether the analysis was performed with or without patient pre-selection. Another possible reason for the difference between the reported prevalence rates and our results might be the higher number of female patients (53.9 % compared with 40.1 % in our study).

However, in all the studies presented here, and in practically all others, a statistically significant correlation was found between gender and EGFR status (mutations are more prevalent in females) and between smoking and EGFR mutation status (mutations are more prevalent in never smokers and former smokers). This is in accordance with our results. It is known that higher EGFR mutation frequencies are observed in female, non-smoking patients of Asian origin [25, 26], reaching more than 60 % in this population. A similar, higher EGFR mutation frequency (52 %) was also found by our study group when only females who are non-smokers were analyzed. The limitation of this finding is that the number of these patients was relatively low (50 patients), but nevertheless, the similarity is obvious.

KRAS mutations are present in many different tumors, including carcinomas of the lung, as KRAS is one of the most commonly mutated genes in human cancer [30]. In lung carcinomas, KRAS mutations are more common in smokers [31–33]. KRAS mutations occur in approximately 30 % of lung adenocarcinomas in Caucasians [30, 34–36] and in approximately 10 % of lung adenocarcinomas in Asians [34, 37]. To date, no effective therapy that targets KRAS has been released, although some therapeutic molecules are currently under investigation [38]. In lung adenocarcinomas, mutations usually occur in codon 12 of exon 2, followed by codon 13 of exon 2 (3-5 %) and rarely (<1 %) in codon 61 of exon 3 [39]. Interestingly, in a Chinese population composed of patients with lung adenocarcinomas who are also smokers, the incidence of KRAS mutations was found to be 14.0 %, while that of non-smokers was 3.4 % [40]. Two studies of Asian patients with NSCLC [41, 42], as well as meta- and pooled analyses [43, 44], demonstrated that the presence of KRAS mutations is a poor prognostic factor in Asians with NSCLC, while its relationship to prognosis in cases of NSCLC in non-Asian patients is still debatable. Additionally, the predictive role of KRAS mutations is not yet completely clear (for review see [45]). Our results demonstrated a slightly higher incidence of KRAS mutations in lung adenocarcinomas than was previously reported for a Caucasian population, which might be due to the smoking habits of this Croatian population. Furthermore, in our population, the correlation of KRAS mutations with smoking proved to be statistically significant even after stratification for gender, which was not the case for the correlation of KRAS mutations with gender after stratification for smoking. The distribution of specific types of mutations in our sample was also similar to previously published data.

At the other end of the mutational landscape of lung adenocarcinomas are ALK gene rearrangements, which occur in 1-6 % of NSCLCs worldwide [46–48]. The meta-analysis conducted by Fengzhi Zhao et al., which included a total of 6950 patients, showed that the prevalence of ALK translocations was 6.8 % in NSCLC patients; this study did not select for ethnicity, but once again demonstrated that ALK translocations are practically mutually exclusive with EGFR and KRAS mutations [49]. They did find that EGFR and ALK alterations occurred simultaneously in 15/6950 patients (0.2 %). Their meta-analysis showed, for the first time, a higher frequency of ALK gene rearrangement in a non-Asian population (8.5 %, 173/2044) without selection for NSCLC, compared with 6.1 % (299/4906) in an Asian population. In a study by Wong et al., the prevalence of ALK gene rearrangement in an East Asian population was 5 % [48]. By contrast, Fu et al. found 44 patients with ALK gene rearrangement, (43/382, 11.3 % of patients with lung adenocarcinoma, and 1/73, 1.3 % of patients with squamous cell lung carcinoma) [50]. The prevalence of ALK gene rearrangement in the Chinese population also differs based on smoking status, as the prevalence is lower in smokers (2.9 %) than in non-smokers (7.2 %) (for review see 42). Based on the fact that IHC and FISH are highly concordant [51, 52], and that IHC was economically feasible, we decided to test the tumors of our patients by IHC only. Our IHC results are in agreement with published results, as are their correlation with gender (higher frequency in females) and smoking status (lower frequency in former smokers and smokers). Smoking and the higher percentage of males in our study group are also the likely causes of the lower incidence of ALK translocations in our population.

Although only 2.4 % of the initial number of patients who were eligible for molecular analysis did not have adequate tissue/tumor cells for any tests, no additional adequate material was available for ALK immunohistochemistry in 8.6 % of the tested samples, which stresses the importance of proper handling of tumor samples in order to have enough for all necessary analyses. This is an important issue that will probably be solved by the introduction of panel tests, in which not only can mutations be analyzed simultaneously, but a smaller amount of material is needed for molecular testing.

## Conclusion

In spite of the considerable published data on the prevalence of EGFR mutations in lung cancer throughout the world, only a few papers present data on EGFR, KRAS and ALK mutations in countries from Central Europe. By contrast, based on the available data, it is usually difficult to determine the actual prevalence of mutations in lung adenocarcinomas since many selection biases occur. These biases primarily concern the clinical stage (patient selection for testing), the histological heterogeneity of the tumors in which mutations are reported, and the testing platforms that are used, which each have a different range of detected mutations. Here, for the first time, we have presented representative data for Croatian patients with lung adenocarcinomas, and we demonstrate a higher prevalence of the specific L858R mutation in the EGFR gene, as well as a higher prevalence of KRAS mutations. By contrast, we show that ALK gene rearrangement was in the range of previously published data compared with data from other European countries. Interestingly, while the mutational status of EGFR and ALK was statistically and significantly correlated with gender as well as with smoking status, KRAS mutations showed a statistically significant correlation only with smoking.

## Additional file

Additional file 1: Table 1. Raw data. (XLSX 30 kb)

## Abbreviations

ALK: Anaplastic lymphoma kinase
ECOG: Eastern Cooperative Oncology Group
EGFR: Epidermal growth factor receptor
FFPE: Formalin fixed paraffin embedded
KRAS: Kirsten rat sarcoma viral oncogene homolog
MGG: May-Grünwald Giemsa
PCR: Polymerase chain reaction
TKI: Tyrosine kinase inhibitor
